# Konnector v2.0: pseudo-long reads from paired-end sequencing data

**DOI:** 10.1186/1755-8794-8-S3-S1

**Published:** 2015-09-23

**Authors:** Benjamin P Vandervalk, Chen Yang, Zhuyi Xue, Karthika Raghavan, Justin Chu, Hamid Mohamadi, Shaun D Jackman, Readman Chiu, René L Warren, Inanç Birol

**Affiliations:** 1 Canada's Michael Smith Genome Sciences Centre, British Columbia Cancer Agency, Vancouver, BC V5Z 4S6, Canada

**Keywords:** Bloom filter, de Bruijn graph, paired-end sequencing, *de novo *, genome assembly

## Abstract

**Background:**

Reading the nucleotides from two ends of a DNA fragment is called paired-end tag (PET) sequencing. When the fragment length is longer than the combined read length, there remains a gap of unsequenced nucleotides between read pairs. If the target in such experiments is sequenced at a level to provide redundant coverage, it may be possible to bridge these gaps using bioinformatics methods. Konnector is a local *de novo *assembly tool that addresses this problem. Here we report on version 2.0 of our tool.

**Results:**

Konnector uses a probabilistic and memory-efficient data structure called Bloom filter to represent a k-mer spectrum - all possible sequences of length k in an input file, such as the collection of reads in a PET sequencing experiment. It performs look-ups to this data structure to construct an implicit de Bruijn graph, which describes (k-1) base pair overlaps between adjacent k-mers. It traverses this graph to bridge the gap between a given pair of flanking sequences.

**Conclusions:**

Here we report the performance of Konnector v2.0 on simulated and experimental datasets, and compare it against other tools with similar functionality. We note that, representing k-mers with 1.5 bytes of memory on average, Konnector can scale to very large genomes. With our parallel implementation, it can also process over a billion bases on commodity hardware.

## Background

If genomes were composed of random sequences, a sequence of length L would be specific enough to describe a locus on a genome of length G when 4^L^>>G. For instance, a typical HiSeq 4000 sequencer (Illumina, San Diego, CA) generates 150 base pair (bp) reads, for which 4^L ^would be more than 80 orders of magnitude larger than the human genome. But, of course, genomes are not random sequences; they have structure, otherwise, we would not be here to write this paper, nor would you be there to read it.

Long read lengths are desirable to reveal structures in genomes of interest. While sequencing technologies from Pacific Biosciences (Menlo Park, CA) and Oxford Nanopore Technologies (Oxford, UK) can generate reads that are several kilo bases (kb) long, their low throughput and high error make them challenging to use in experiments that interrogate large targets.

Many experimental designs with short sequencing data use a paired-end tag (PET) sequencing strategy, where short sequences are determined from both ends of a DNA fragment. These PET sequences are then associated in downstream analysis to resolve structures as long as fragment lengths. Typically, these fragments are less than 1 kb, and ideally have unimodal length distributions. To resolve even longer structures, there are specialized library preparation protocols, such as Nextera and Moleculo from Illumina and GemCode from 10X Genomics (Pleasanton, CA).

In this study, we focus on the PET reads. We describe Konnector v2.0, a tool that uses the coverage redundancy in a high-throughput sequencing experiment to reconstruct fragment sequences (pseudo-reads). Optionally, it also extends those fragment sequences in 3' and 5' directions, as long as the extensions are unambiguous. The tool builds on our earlier implementation [1] that filled in the bases of the sequence gap between read pairs by navigating a de Bruijn graph [2]. Konnector represents a de Bruijn graph using a Bloom filter [3], a probabilistic and memory-efficient data structure.

The utility of long pseudo-reads has been demonstrated before [4], and forms the backbone of some *de novo *assembly tools [5]. Long pseudo-reads can be generated by merging overlapping PETs [6,7], or by localizing the sequence assembly problem around PETs [8,9]. Our focus in this study is the latter problem.

For example, the ELOPER algorithm [8] identifies read pairs that share an overlap in both reads simultaneously, and uses these overlaps to generate "elongated paired-end reads". The GapFiller algorithm [9], on the other hand, formulates this problem as a collection of seed-and-extend local assembly problems. The latter concept has also been implemented within the MaSuRCA *de novo *assembly pipeline [5], a wrapper around the Celera Assembler software [10].

We benchmark Konnector v2.0 on simulated datasets, compare its performance against ELOPER [8], GapFiller [9], and a similar tool within MaSuRCA [5]. We demonstrate its utility for assembly finishing problems and variant calling. With its frugal memory use and algorithm implementation, we show that Konnector v2.0 can handle large sequence datasets with over a billion reads from Gbp scale genomes in a timely manner. Furthermore, we note that it consistently provides highly accurate results for a range of targets.

## Implementation

**Figure 1:**
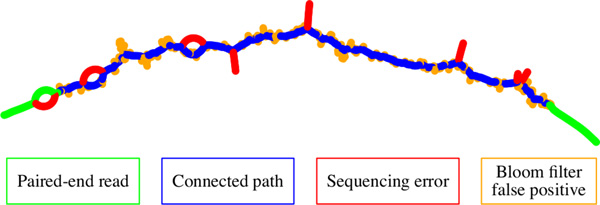
**A connecting path between two non-overlapping paired-end sequencing reads within a de Bruijn graph**. Konnector joins the sequence provided by the input paired-end reads (green) by means a graph search for a connecting path (blue). Sequencing errors in the input sequencing data produce bubbles and branches in the de Bruijn graph of up to k nodes in length (red). Bloom filter false positives produce additional branches (yellow) with lengths that are typically much shorter than the error branches.

**Figure 2:**
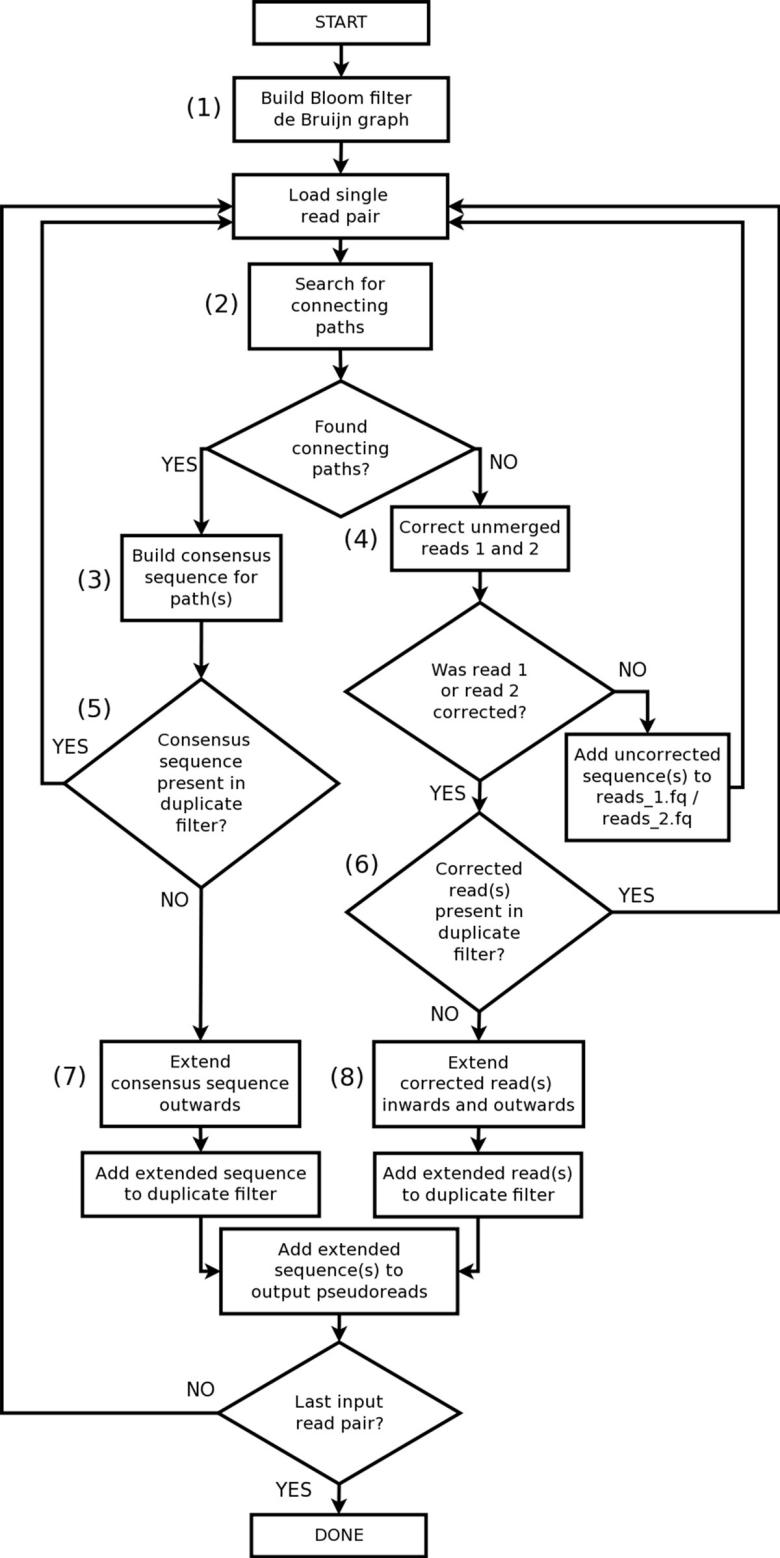
**The Konnector2 algorithm**. (1): The algorithm builds a Bloom filter representation of the de Bruijn graph by loading all k-mers from the input paired-end sequencing data. (2): For each read pair, a graph search for connecting paths within the de Bruijn graph is performed. (3): If one or more connecting paths are found, a consensus sequence for the paths is built. (4): If no connecting paths are found, error-correction is attempted on reads 1 and 2. (5) and (6): the algorithm queries for the existence of either the consensus connecting sequence or the error-corrected reads in the "duplicate filter". The duplicate filter is an additional Bloom filter, separate from the Bloom filter de Bruijn graph, which tracks the parts of the genome that have already been assembled. (7) and (8): If one or more of the k-mers in the query sequence are not found in the duplicate filter, the sequence is extended outwards in the de Bruijn graph, until either a dead end or a branching point is encountered in the graph. Finally, the extended sequences are written to the output pseudo-reads file.

Konnector creates long pseudo-reads from paired-end sequencing reads (Figure 1) by searching for connecting paths between read pairs using a Bloom filter representation of a de Bruijn graph. In addition to connecting read pairs, Konnector v2.0 can also extend connected or unconnected sequences by following paths from the ends of sequences up to the next branching point or dead end in the de Bruijn graph. When the sequence extension feature of Konnector v2.0 is enabled, an additional Bloom filter is employed to avoid the production of an intractable quantity of duplicate sequences. Figure 2 provides a flowchart overview of the Konnector 2.0 algorithm.

### Bloom filter de Bruijn graph

As the throughput of the Illumina platforms increased rapidly to generate up to 1Tb in a six-day run with the HiSeq SBS V4 Kits, one important concern for pseudo-read generating tools is their computational efficiency. In related problems, bioinformatics tools have used strategies such as parallel computing [11,12], FM indexing [13,14], and compressed data structures [15] for handling big data.

To fit large assembly problems in small memory, one recent approach has been the use of Bloom filters [16,3] to represent de Bruijn graphs, as demonstrated by the Minia assembler [17]. Konnector adopts a similar approach. Briefly, a Bloom filter is a bit array that acts as a compact representation of a set, where the presence or absence of an element in the set is indicated by the state of one or more bits in the array. The particular position of the bits that correspond to each element is determined by a fixed set of hash functions. While Bloom filters are very memory-efficient, the principal challenge of developing Bloom filter algorithms is in dealing with the possibility of *false positives*. A false positive occurs when the bit positions of an element that is not in the set collide with the bit positions of an element that *is *in the set. In the context of Bloom filter de Bruijn graphs, false positives manifest themselves as false branches, as depicted by the yellow nodes in Figure 1.

In the first step of the algorithm (Figure 2, step (1)), the Bloom filter de Bruijn graph is constructed by shredding the input reads into k-mers, and loading the k-mers into a Bloom filter. To diminish the effect of sequencing errors at later stages of the algorithm, k-mers are initially propagated between two Bloom filters, where the first Bloom filter contains k-mers that have been seen at least once, and the second Bloom filter contains k-mers that have been seen at least twice. At the end of k-mer loading, the first Bloom filter is discarded, and the second Bloom filter is kept for use in the rest of the algorithm. We note here that only the k-mers of the input reads, corresponding to the nodes in the de Bruijn graph, are stored in the Bloom filter whereas there is no explicit storage of edges. Instead, the neighbours of a k-mer are determined during graph traversal by querying for the presence of all four possible neighbours (i.e. single base extensions) at each step.

### Searching for connecting paths

In a second pass over the input sequencing data, Konnector searches for connecting paths within the de Bruijn graph between each read pair (Figure 2, step (2)). The graph search is initiated by choosing a start k-mer in the first read and a goal k-mer in the second read, and is carried out by means of a depth-limited, bidirectional, breadth-first search between these two k-mers.

The start and goal k-mers are selected to reduce the probability of dead-end searches due to sequencing errors or Bloom filter false positives. First, the putative non-error k-mers of each read are identified by querying for their existence in the Bloom filter de Bruijn graph. (Recall that after the loading stage, this Bloom filter only contains k-mers that occur twice or more.) Next, the algorithm attempts to find a consecutive run of three non-error k-mers within the read, and chooses the k-mer on the distal end (i.e. 5' end) of the run as the start/goal k-mer. This method ensures that if the chosen start/goal k-mer is a Bloom filter false positive, the path search will still proceed through at least two more k-mers instead of stopping at a dead end. In the likely case that there are multiple runs of "good" k-mers within a read, the run that is closest to the 3' (gap-facing) end of the read is chosen, in order reduce the depth of subsequent path search. In the case that there are no runs of three good k-mers, the algorithm falls back to using the longest run found (i.e. two k-mers or a single k-mer).

Once the start and goal k-mers have been selected, Konnector performs the search for connecting paths. In order to maximize the accuracy of the sequence connecting the reads, it is important for the algorithm to consider *all *possible paths between the reads, up to the depth limit dictated by the DNA fragment length. For this reason, a breadth-first search is employed rather than a shortest path algorithm such as Dijkstra or A*. Konnector implements a bidirectional version of breadth-first search, which improves performance by conducting two half-depth searches, and thus reducing the overall expansion of the search frontier. The bidirectional search is implemented by alternating between two standard breadth-first searches that can "see" each other's visited node lists. If one search encounters a node that has already been visited by the other search, the edge leading to that node is recorded as a "common edge", and the search proceeds no further through that particular node. As the two searches proceed, all visited nodes and edges are added to a temporary, in-memory "search graph". This facilitates the final step, where the full set of connecting paths are constructed by performing an exhaustive search both backwards and forwards from each common edge towards the start and goal k-mers, respectively.

If the search algorithm finds a unique path between the start and goal k-mers, then the path is converted to a DNA sequence, and is used to join the read sequences into a single pseudo-read. In the case of multiple paths, a multiple sequence alignment is performed, and the resulting consensus sequence is used to join the reads instead (Figure 2, step (3)). In order to fine-tune the quality of the results, the user may specify limits with respect to the maximum number of paths that can be collapsed to a consensus and/or the maximum number of mismatches that should be tolerated between alternate paths.

### Extending connected and unconnected sequences

Konnector v2.0 introduces a new capability to extend both connected and unconnected sequences by traversing from the ends of sequences to the next branching point or dead-end in the de Bruijn graph (Figure 2, steps (7) and (8)). If a read pair is successfully connected, the algorithm will extend the pseudo-read outwards in both directions; if the read pair is not successfully connected, each of the two reads will be extended independently, both inwards and outwards. The extensions are seeded in the same manner described above for the connecting path searches; a putative non-error k-mer is selected near the end of the sequence, and following two consecutive non-error k-mers if possible.

The extension of connected reads or unconnected reads that are contained within the same linear path of the de Bruijn graph results in identical sequences. For this reason, the algorithm uses an additional Bloom filter to track the k-mers of sequences that have already been assembled. (Hereafter this Bloom filter will be referred to as the "duplicate filter" in order to reduce confusion with the Bloom filter de Bruijn graph.)

The logic for tracking duplicate sequences differs for the cases of connected and unconnected read pairs. In the case of connected reads, only the k-mers of the connecting sequence are used to query the duplicate filter (Figure 2, step (5)). By virtue of being present in the Bloom filter de Bruijn graph, the connecting k-mers are putative non-error k-mers that have occurred at least twice in the input sequencing data, and thus a 100% match is expected in the case that the genomic region in question has already been covered. If one or more k-mers from the connecting sequence are not found in the duplicate filter, the pseudo-read is kept and is extended outwards to its full length (Figure 2, step (7)). The k-mers of the extended sequence are then added to the duplicate filter, and the sequence is written to the output pseudo-reads file.

In the case of unconnected reads, the reads must first be corrected prior to querying the duplicate filter (Figure 2, step (4)). This is done by first extracting the longest contiguous sequence of non-error k-mers within the read, where k-mers that are present in the Bloom filter de Bruijn graph are considered to be putative non-error k-mers. An additional step is then performed to correct for recurrent read-errors that may have made it past the two-level Bloom filter. Starting from the rightmost k-mer of the selected subsequence, the algorithm steps left by k nodes, aborting the correction step if it encounters a branching point or dead-end before walking the full distance. As the longest branch that can be created by a single sequencing error is k nodes, this navigates out of any possible branch or bubble created by an error (red nodes of Figure 1). Finally, the algorithm steps right up to (k+1) nodes to generate a high confidence sequence for querying the duplicate filter. The second rightward step stops early upon encountering a branching point or dead-end, but any sequence generated up to that point is kept, and is still used to query the duplicate filter. Following error correction, the subsequent steps for handling unconnected reads are similar to the case for connected reads. If the high confidence sequence contains k-mers that are not found in the duplicate filter, the sequence is extended to its full length, added to the duplicate filter, and written to the output pseudo-reads file.

Finally, some additional look-ahead logic is employed in the extension algorithm to handle the common cases of false positive branches and simple bubbles created by heterozygous SNPs. All branches shorter than or equal to three nodes in length are assumed to be false positive branches and are ignored during extension. Upon reaching a fork with two (non-false-positive) branches, a look-ahead of (k+1) nodes is performed to see if the branches re-converge. If so, the bubble is collapsed and the extension continues.

## Results and discussion

### Read-elongation tools comparison

To evaluate Konnector v2.0, we performed a comparison with several other read-elongation tools: ELOPER [8], GapFiller [9], the MaSuRCA super-reads module [5], and the previously published version of Konnector [1].

ELOPER v1.2 (ELOngation of Paired-End Reads) [8] operates by calculating *gapped overlaps *between read pairs, where a gapped overlap requires simultaneous overlap of both reads across two read pairs. The main idea of the algorithm is that overlaps across read pairs yield higher-confidence sequence extensions than overlaps between individual reads alone. The program produces extended paired-end reads as output and single-end pseudo-reads in cases where paired reads can be extended far enough to overlap with their mates. The all-by-all computation of gapped overlaps between read pairs is realized using a hash table-based approach.

GapFiller v2.1.1 [9] fills the sequencing gap between paired-end reads using a seed-and-extend approach, where each input read is considered in turn as a seed. Reads are iteratively extended towards their mates by identifying overlapping reads, building a consensus sequence for the extension, and then repeating the process with extended sequence. GapFiller uses the eventual overlap of an extended read with its mate as a correctness check, and chooses not to continue the extension beyond the fragment length in favour of higher confidence results. The algorithm for detecting overlaps is implemented by computing some *fingerprint *values for the prefixes and suffixes of each read, and storing the mapping between fingerprints and reads in a hash-table. In order to calculate the consensus sequences during extension, the full set of input read sequences is stored in memory using a compressed 2-bit representation.

MaSuRCA v2.2.1 [5] is an extension of the CABOG overlap-layout-consensus assembler [18] that preprocesses the input short sequencing reads to generate a highly-reduced set of "super-reads" for input to the Celera assembler [10]. Much like the extension feature of Konnector v2.0, the super-reads are generated by extending the reads outward to the next branching point or dead-end within a de Bruijn graph. These "k-unitig" sequences are then joined by spanning read pairs or bridging single-end reads, in cases where such links are unambiguous.

The previously published version of Konnector [1] uses the same concept for connecting read pairs as Konnector v2.0, but does not include the sequence extension or duplicate filtering logic. Its output format is most similar to GapFiller, in the sense that it generates one fragment-length sequence for each successfully connected read pair.

We compared the performance and results of the tools across four paired-end sequencing data sets from organisms with a wide range of genome sizes: *E. coli, S. cerevisiae, C. elegans*, and *H. sapiens *(Table 1). The *E. coli *data set consists of 100 bp synthetic reads generated with the pIRS read simulator [19] using a 0.1% error rate, 50x coverage, and an insert size of 400 ± 50 bp, while the other three data sets are experimental paired-end Illumina sequencing data with coverage levels ranging from 26x to 76x.

**Table 1:** Datasets analyzed.

Organism	Genome Size	NGS data source	Read length (bp)	Read pairs (M)	Fragment size (bp)	Fold coverage
*E.coli* K-12	5 Mbp	Simulated	PE100	1.2	400	50X

*S. cerevisiae*	12 Mbp	Experimental SRA:ERR156523	PE100	1.6	300	26X

*C.elegans*	97 Mbp	Experimental SRA:ERR294494	PE100	44.7	450	89X

*H.sapiens* NA19238	3 Gbp	Experimental SRA:ERR309932	PE250	457.0	550	76X

For each combination of data set and tool, we measured running time, peak memory usage, N50 length of the output pseudo-reads, sum length of the misassembled pseudo-reads, and percent coverage of the reference genome (Table 2). The N50 length was calculated using the QUAST [20] assembly assessment tool, except for the human data set where the 'abyss-fac' utility (ABySS v1.5.2 [11]) was used instead. The "Misassembled Reads Length" column of Table 2 was also calculated by QUAST, and reports the sum length of all pseudo-reads that had split alignments to the reference with distance greater 1 kb, overlap greater 1 kb, or mappings to different strands/chromosomes. We found that QUAST was not able to scale to an analysis of the human Konnector and Konnector v2.0 pseudo-reads, and so those results were omitted from Table 2. Finally, the genome coverage results were calculated by aligning the pseudo-reads to the reference with bwa mem v0.7.12 [21], with the multimapping option (-a) turned on, and then using the resulting BAM file as input to the bedtools v2.17.0 [22] 'genomecov' command.

**Table 2:** Comparative analysis of read elongation tools.

	Running time (hms)	Peak memory (MB)	N50 (bp)	Misassembled Reads Length (bp)	Percent genome coverage
*E. coli *(synthetic)					

ELOPER	10m46s	19013	501	16146	**100.00**

GapFiller	32m39s	476	396	14103	**100.00**

MaSuRCA super-reads	**2m56s**	4669	**54103**	**0**	**100.00**

Konnector (k = 50)	6m15s	**81**	406	3133	**100.00**

Konnector 2 (k = 70)	5m0s	100	32012	276	99.99

*S. cerevisiae*					

ELOPER	97h55m23s	37119	144	71426	**99.32**

GapFiller	3h13m18s	666	332	96869	97.04

MaSuRCA super-reads	**4m2s**	5294	1684	129828	98.67

Konnector (k = 50)	8m34s	**231**	315	75435	97.94

Konnector 2 (k = 40)	7m1s	232	**4690**	**67505**	98.99

*C. elegans*					

ELOPER	exceeds available memory (120GB)				

GapFiller	exceeds available memory (120GB)				

MaSuRCA super-reads	**2h2m17s**	80742	2554	1925740	**99.98**

Konnector (k = 55)	5h5m21s	**1954**	475	NA	99.80

Konnector 2 (k = 80)	3h30m23s	2193	**6232**	**837480**	99.88

*H. sapiens (NA19238)*					

ELOPER	not attempted				

GapFiller	not attempted				

MaSuRCA super-reads	exceeds available memory (120 GB)				

Konnector (k = 150)	4d9h15m48s	**410381**	556	NA	**94.15**

Konnector 2 (k = 180)	**20h47m24s**	471905	**3051**	NA	94.01

**Figure 3:**
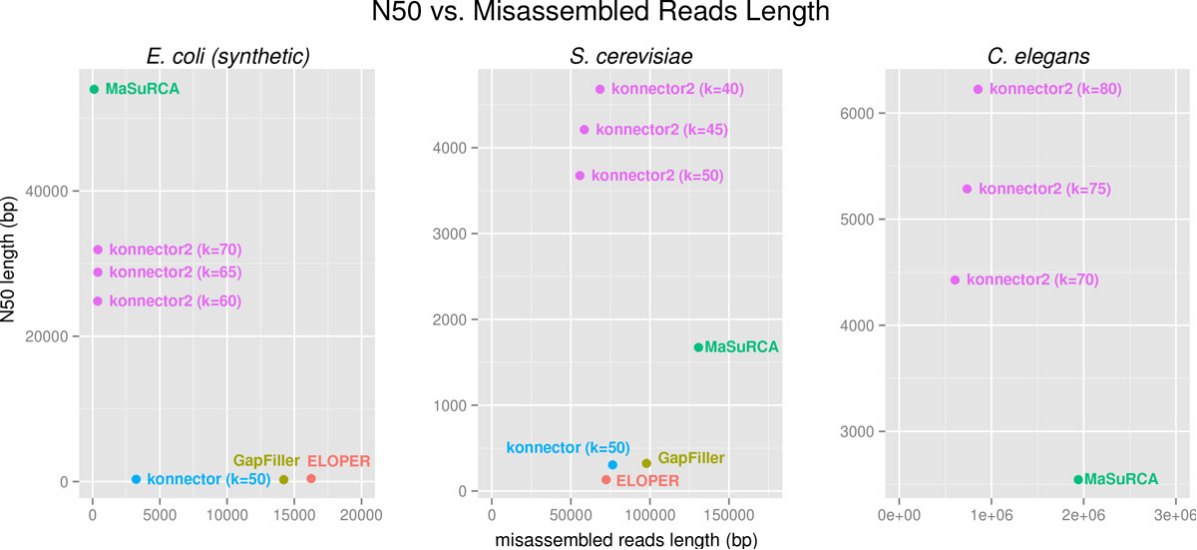
**Comparison of pseudo-read tools by N50 and misassembled reads length**. Results are shown for Konnector v2.0 and three other pseudo-read-generating tools across *E. coli *(synthetic), *S. cerevisiae*, and *C. elegans *sequencing data sets. The "misassembled reads length" on the x-axis of each plot denotes the sum length of all pseudo-reads reported as misassembled by QUAST. MaSuRCA performs best on the synthetic *E. coli *data set, producing the highest N50 and creating no misassemblies. However, on the experimental *S. cerevisiae *and *C. elegans *data sets, Konnector v2.0 outperforms the other tools in terms of both N50 and misassembled sequence, for a range of k-mer lengths.

The Konnector and Konnector v2.0 jobs for the comparison were run across a range of k-mer lengths to achieve the best possible results. For the previous version of Konnector, the run with the highest percentage of connected read pairs was selected, whereas for Konnector v2.0, a k-mer size was selected that provided a favourable combination of both N50 and misassembled reads length (Figure 3).

From the results of Table 2, we observe that MaSuRCA was generally the fastest tool. While Konnector and Konnector v2.0 showed competitive run times, ELOPER and GapFiller were notably slower, and did not scale well to larger data sets. In the category of memory usage, both versions of Konnector outperformed the competitors by more than an order of magnitude due to their use of Bloom filters rather than hash tables.

The N50 and Misassembled Reads Length results from Table 2 are plotted in Figure 3, with additional data points shown for alternate runs of Konnector v2.0 with different k-mer sizes. The plots show that Konnector 2 generated the longest pseudo-reads and the least misassembled sequence for the experimental *S. cerevisiae *and *C. elegans *data sets, while MaSuRCA generated the longest and most accurate pseudo-reads for the synthetic *E. coli *data set.

Working with a maximum of 120 GB available memory on any single machine, Konnector and Konnector v2.0 were the only tools that could be run on the largest of the four data sets in Table 2 (*H. sapiens*). One of the main advantages of Konnector for this data set was the ability to split work across machines. This was accomplished by first building a reusable Bloom filter file with the companion 'abyss-bloom' utility (ABySS v1.5.2), and then sharing this file across 20 parallel Konnector jobs, each processing a disjoint subset of the paired-end reads. The two-level Bloom filter size was 40 GB, and each of the jobs was run on a machine with 12 cores and 48 GB RAM. The wall clock time for the job was less than 24 hours, and the aggregate memory requirement for job was just under 0.5TB. We note that the larger memory usage of Konnector v2.0 is due to the use of an additional Bloom filter for tracking duplicate sequences. The large improvement in running time between Konnector and Konnector v2.0 on the *H. sapiens *data set is due primarily to the introduction of multithreaded Bloom filter construction in Konnector v2.0.

### Sealer: a Konnector-based gap-closing application

A natural application to Konnector includes automated finishing of genomes, by systematically targeting all regions of unresolved bases, or gaps, in draft genomes of wide-ranging sizes. This is accomplished by first identifying these scaffold gaps, deriving flanking sequences on the 5' and 3' ends of each gap, running Konnector with comprehensive short read data set, and patching the gaps by placing successfully merged sequences in those regions. We have developed a stand-alone utility called Sealer for this specific application [23].

To test the utility of Konnector for filing scaffold gaps, we ran Sealer on an ABySS *E. coli *genome assembly (5 Mbp) and, to assess the scalability of the approach, on an ABySS *H. sapiens *(3.3 Gbp) draft assembly of next-generation Illumina sequences (SRA:ERR309932) derived from the 1000 Genomes Project (individual NA19238) (Table 3). For *E. coli*, we were able to successfully close all but one gap using a single k-mer size of 90 bp. On the human assembly, gaps were closed with Sealer using 31 k-mers (250 - 130 bp, decrementing by 10, and 125 - 40 bp, decrementing by 5; parameters for Konnector were -B 1000 -F 700 -P10), and compared the result to two similar tools GapFiller (v1.10) [24] and SOAPdenovo2 GapCloser (v1.12) [25]. Default settings were used for both tools in our tests, maximizing the number of compute threads, when needed (-t 16 for GapCloser on the human data set). On the *H. sapiens *draft assembly GapFiller was manually stopped after running for over 350 hours (approximately 14 days) without completion or output. All Sealer processes were executed on a 12-core computer running CentOS 5.4 with two Intel Xeon X5650 CPUs @ 2.67 GHz and 48 GB RAM. GapFiller and GapCloser were benchmarked on a machine using CentOS 5.10 with 16 cores @ 2.13 GHz, 125 GB RAM. The GapCloser run on the *H. sapiens *data ran on a CentOS 5.9 with 16 cores @ 2.13 GHz and 236 GB RAM to allow for its high memory requirement. We also compared the results of Sealer with two versions of Konnector on the *E. coli *and *H. sapiens *dataset, and noticed a marked improvement in speed of execution: ~12 h compared to ~29 h runs on human data with Konnector v2.0 and Konnector v1.0, respectively. We also noted improved sensitivity in Sealer results, when used in conjunction with Konnector v2.0 (6,566 or 2.8% more gaps closed). To test the limits of scalability of Konnector, we applied Sealer on a draft white spruce genome assembly of length 20 Gbp [26] (data not shown).

**Table 3:** Performance evaluation of Sealer and other gap-filling applications for finishing draft genomes.


**Draft genome species**	**Total gaps**	**Software**	**Gaps completely closed**	**% Success**	**Wall clock time (hh:mm)**	**Memory (GB)**

*E. coli*	18	Sealer K2	**17**	**94.4**	00:01	0.5

		Sealer K1	**17**	**94.4**	00:20	0.5

		GapCloser	2	11.1	**00:05**	25.7

		GapFiller	15	83.3	00:43	**0.4**

*H. sapiens*	237,406	Sealer K2	**127,242**	**53.6**	**12:09**	**22.2**

		Sealer K1	120,676	50.8	29:19	**22.2**

		GapCloser	116,297	48.9	83:15	178.1

		GapFiller	Incomplete. Terminated after 353 hours.			

### KVarScan: a Konnector-based method for indel detection

Konnector long pseudo-reads can potentially improve the sensitivity of existing variant detection pipelines. To explore this idea, we conducted an experiment where we detected insertions and deletions (indels) using VarScan [27] 'pileup2indel' (version 2.3.7 with default parameters), and compared the results when using either regular reads or Konnector pseudo-reads as input. We refer to the two protocols as VarScan and KVarScan, respectively. For KVarScan, Konnector reads were generated by running Konnector on regular reads for a range of k-mer sizes from 90 bp to 30 bp, with a step size of 10 bp. Starting with the largest k-mer size of 90 bp, left over read pairs that were not connected were used for the next run of Konnector with the next smaller k-mer size. After running Konnector with a k-mer size of 30 bp, any remaining unconnected reads were used concurrently with the connected reads as input to the VarScan run. All connected reads output from the -p/--all-paths and -o/--output-prefix parameters of Konnector were used. The other parameters used for Konnector included: max path set to 4 (-P), max mismatches set to nolimit (-M), path identity set to 98 (-X), max branches set to 100 (-B), max fragment set to 525 (-F). Prior to the Konnector runs, a Bloom filter for each k-mer size was built using the abyss-bloom utility with the trim quality (-q) set to 15 and levels (-l) set to 2.

We performed a comparison of the VarScan and KVarScan methods on synthetic human data from chromosome 10 by simulating indels in the size range of 10 - 200 bp on hg19 using RSVsim v1.2.1 [28]. A final number of 224 insertions (10 - 192 bp) and 216 deletions (10-144 bp) were generated. We used pIRs v1.1.1 [19] to generate a diploid human chromosome 10 sequence, and combined it with the rearranged sequence to simulate a 30x coverage library of 100 bp Illumina PET reads. The average insert size was set at 400 bp; default parameters were used otherwise.

**Figure 4:**
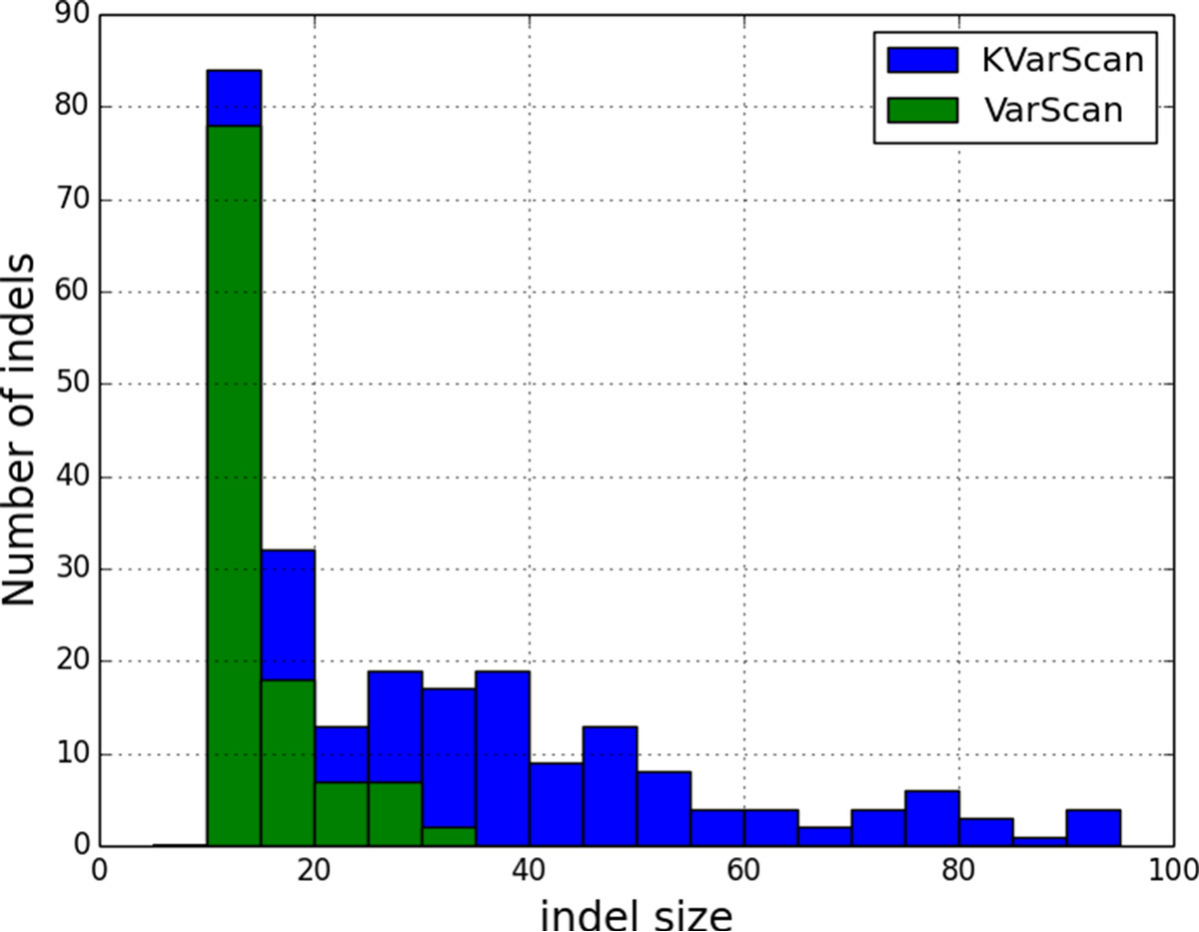
**Indels detected by VarScan using unaltered reads ("VarScan") or Konnector long pseudo-reads ("KVarScan") as input**. Results are shown for synthetic read data generated from hg19 chromosome 10 and containing 440 simulated indels. The indels detected by VarScan (green) range from 10 bp to 30 bp, whereas the indels detected by KVarScan (blue) range from 10 bp to 99 bp.

Both VarScan and KVarScan were able to detect small indels as short as 10 bp, the shortest available in the simulated data. However, the maximum size of indels detected by VarScan was 30 bp, while it was 99 bp for the KVarScan protocol. As illustrated by the distributions of the sizes of indels detected in Figure 4, we note that the use of long pseudo-reads generated by Konnector expands the range detection for VarScan. Hence, long pseudo-reads may find an application for profiling cancer genomes and other genomes that harbour structural variations that would otherwise be missed by shorter sequence reads.

## Conclusions

Long reads are highly desirable for both *de novo *assembly applications and reference-based applications such as variant calling. While long read sequencing technologies such Pacific Biosciences (Menlo Park, CA) and Oxford Nanopore Technologies (Oxford, UK) have yet to hit the mainstream, bioinformatics algorithms continue to be developed to better exploit the sequence and distance information captured by Illumina paired-end sequencing reads, currently ranging in length from 150 - 300 bp and spanning DNA fragments with sizes of 300 - 1000 bp.

In this paper we have presented Konnector v2.0, a tool for producing long "pseudo-reads" from Illumina paired-end libraries. While many tools exist to merge overlapping paired-end reads (e.g. [6,7]), our software addresses the more challenging problem of filling the sequencing gap between non-overlapping reads. Konnector accomplishes this by building a compact, Bloom filter based representation of a de Bruijn graph and performing a constrained path search between each pair of reads within the graph. Konnector v2.0 introduces a significant improvement to the algorithm by additionally extending sequences outwards within the de Bruijn graph, up to the point where such extensions are unambiguous. It also keeps the functionality of Konnector v1.0, as an option.

In a comparison of Konnector v2.0 against several similar tools, we have demonstrated that the software generates pseudo-reads with high accuracy, high yield, low memory usage, and fast run times. Owing to its use of a Bloom filter de Bruijn graph, Konnector was the only tool able to process 76x human sequencing data on a set of computing nodes with 48 GB of RAM, and was able to do so in under 24 hours.

While the long pseudo-read generating tools were all reported for their utility in *de novo *assembly applications in earlier studies [6-8], we demonstrated the utility of our tool on two novel uses cases: assembly finishing and variant detection. With its scaling properties and broad applications, we think Konnector will be an enabling technology in many genomics studies.

## Availability and requirements

**Project name: **Konnector

**Project home page: **http://www.bcgsc.ca/platform/bioinfo/software/konnector

**Source code for version in evaluated in paper: **https://github.com/bcgsc/abyss/tree/konnector2-prerelease

**Operating system(s): **Unix

**Programming language: **C++

**Other requirements: **Boost graph library, Google sparsehash library is recommended

**License: **Free for academic use under the British Columbia Cancer Agency's academic license

**Any restrictions to use by non-academics: **Contact ibirol@bcgsc.ca for license

## List of abbreviations used

ABySS: Assembly By Short Sequences; CABOG: Celera Assembler with the Best Overlap Graph; ELOPER: Elongation of Paired-end Reads; MaSuRCA: Maryland Super-Read Celera Assembler.

## Competing interests

The authors declare that they have no competing interests.

## Authors' contributions

BV implemented the search and extension algorithms for Konnector v2.0, and wrote descriptions of the algorithm and tools comparison. CY conducted analyses for the tools comparison and for Sealer. KR ran QUAST evaluations and other exploratory data analyses. ZX and RC did the analysis and writing for the KVarScan application. HM and JC provided improved Bloom filter algorithms and implementations. SDJ implemented the Bloom filter class and the de Bruijn graph interface for Konnector, and also implemented algorithmic improvements for the new version of Sealer. RLW did analysis and writing for the Sealer section, ran jobs for the tools comparison, and oversaw the planning and organization the paper. IB designed the algorithms for Konnector v2.0 and oversaw the development, evaluation, and manuscript preparation.

## Declarations

The authors thank the funding organizations, Genome Canada, British Columbia Cancer Foundation, and Genome British Columbia for their partial support of the publication. Research reported in this publication was also partly supported by the National Human Genome Research Institute of the National Institutes of Health under Award Number R01HG007182. The content is solely the responsibility of the authors and does not necessarily represent the official views of the National Institutes of Health or other funding organizations.

This article has been published as part of BMC *Medical Genomics *Volume 8 Supplement 3, 2015: Selected articles from the IEE International Conference on Bioinformatics and Biomedicine (BIBM 2014): Medical Genomics. The full contents of the supplement are available online at http://www.biomedcentral.com/bmcmedgenomics/supplements/8/S3.
